# The Impact of Affective Context on Autobiographical Recollection in Depression

**DOI:** 10.1177/2167702617740672

**Published:** 2017-11-16

**Authors:** Caitlin Hitchcock, Ann-Marie J. Golden, Aliza Werner-Seidler, Willem Kuyken, Tim Dalgleish

**Affiliations:** 1 Medical Research Council (MRC) Cognition and Brain Sciences Unit, Cambridge University; 2 Cambridgeshire and Peterborough National Health Service Foundation Trust, Cambridge, England; 3 The Black Dog Institute, University of New South Wales; 4 University of Oxford

**Keywords:** autobiographical memory, depression, emotional processing biases, open data, open materials

## Abstract

Across two studies we investigated the influence of contextual cues on autobiographical memory recall. In Study 1, participants (*N* = 37) with major depressive disorder, in episode or in varying degrees of remission, were administered a Negative Autobiographical Memory Task (NAMT) that required them to retrieve *negatively* valenced memories in response to *positive* cue words (a positive context). We reasoned that increased depression symptom severity would be associated with a reduced ability to override priming from this disadvantageous context. Consequently, we hypothesized that increased depressive severity would counterintuitively be associated with *reduced* negativity ratings for retrieved personal memories to positive cues on the NAMT. This hypothesis was supported. Study 2, using a community sample (*N* = 63), demonstrated that a similar reduction in memory negativity was observed in individuals with lower working memory capacity—an index of executive control. Implications for autobiographical memory and executive training paradigms for depression are discussed.

The ability to retrieve autobiographical memories pertinent to current goals and circumstances is a core component of everyday cognition. Personal memories are an important ingredient in successful problem solving, future planning, social discourse, and mood regulation (Addis, Hach, & Tippett, 2016; Goddard, Dritschel, & Burton, 1996; Jing, Madore, & Schacter, 2016). When seeking to recollect the autobiographical past in the service of such activities, the nature of what we remember not only is dictated by our explicit retrieval intentions but also is shaped by the contextual cues in the environment where recollection takes place (e.g., Ball & Hennessy, 2009; Mace, 2005). These contextual priming effects generally serve to maximize the relevance of our memories to current circumstances. However, in some situations contextual cues can direct recollection toward mnemonic content that is discrepant with our intentions and thereby unwanted (Hertel & Brozovich, 2010). In such circumstances, the ability to override the influence of context is critical for the successful pursuit of our task goals.

There is accumulating evidence that people suffering from depression have a particular difficulty in overriding contextual influences across different domains of cognition including autobiographical recollection (e.g., Bargh & Tota, 1988; Dalgleish et al., 2007; Williams et al., 2007). For example, in a previous study (Dalgleish et al., 2007) we showed that depressed individuals’ ability to successfully recall specific personal memories of emotional events lasting a day or less was markedly reduced when those memories were cued by words that contextually primed longer periods of time (e.g., *holiday, adolescence*). Similarly, depressed individuals selectively remember events from their personal past that are hedonically congruent with their depressed mood, despite this being at odds with current task goals (e.g., Clark & Teasdale, 1982). In an everyday setting, an overriding influence of contextual cues could mean that depressed individuals will find it difficult to recall positive or neutral events in the face of negative intrinsic cues, such as negative thoughts. This may impair everyday adaptive functioning such as problem solving if the individual is unable to override negative contextual cues (e.g., thoughts of failure, hopelessness) to recall a positive memory of a previous occasion when they had successfully solved a similar problem.

Here we investigate the potency of affective contextual priming effects to override retrieval goals in depression. We reasoned that particularly compelling evidence for such priming would be provided if we could manipulate the retrieval context such that depressed individuals actually inadvertently recalled personal memories that were more positively valenced than the retrieval instructions demanded, relative to those who are less depressed.

To examine this question we designed a laboratory paradigm—the Negative Autobiographical Memory Task (NAMT). In this task, participants are asked to recall a negative memory, primed by a cue word. Ostensibly, the NAMT is a paradigm that depressed participants should perform well at because the aim of the task is to retrieve memories of emotionally *negative* past events (i.e., mood congruent recall). It is well established that for those with depression such negative memories are relatively more easily brought to mind (see Blaney, 1986). Consequently, pursuit of a task goal to explicitly recollect such material should be facilitated by this memory bias. However, for half of the trials on the NAMT we presented participants with positive cue words (e.g., *joy*) to prompt negative autobiographical recollection; that is, the *negative* autobiographical memory recall was primed using a *positive* contextual cue.

To the extent that recalled content is influenced by the nature of these positive contextual cues, we reasoned that the retrieved memories would be rendered more positive, or at least less negative, in valence even though the task goal was to retrieve negative material (M. A. Conway & Pleydell-Pearce, 2000). Our rationale was that the positive cues would bias the search of the autobiographical database toward commencing at what Lyubomirsky, Caldwell, and Nolen-Hoeksema (1998, p. 175) term a positive “starting point” on a “hedonic continuum” within the memory space that spans the repositories of positive and negative personal memories. As a result, although the memory search would almost always eventually shift to interrogate the negative memory domain in line with the explicit task instructions, the negative memories that are eventually retrieved would be sourced from points closer on this hedonic continuum to the positive memory repository and would thus be less negative in valence.

Ostensibly, this effect would be enhanced when the executive control skills required to offset it are impaired by depression (for meta-analysis, see McDermott & Ebmeier, 2009). As in other domains of cognition, we have some capacity to override or offset the influence of maladaptive context and thus to regulate, inhibit, and guide autobiographical recollection using effortful processes of executive control (Levy & Anderson, 2008; Williams, 2006). Thus, the reduction in executive control that is associated with increasing depressive severity would be predicted to reduce the ability to resist the priming effect (Dalgleish et al., 2007; McDermott & Ebmeier, 2009) of the positive cue and impede the ability to shift the memory search toward to the negative end of the autobiographical continuum.

In sum, if depression severity is associated with a relatively weaker ability to override the effects of priming from contextual cues, then increased severity of depression should paradoxically be associated with the recollection of *less* strongly negatively valenced memories on the NAMT.

## Study 1: NAMT Performance in Individuals With Clinical Depression

To test this prediction, after successful validation and piloting of the NAMT in a dysphoric sample (see the method section), in Study 1 we presented the NAMT to participants with a diagnosis of recurrent major depressive disorder (MDD), some of whom were in episode and some in partial remission, and who therefore presented with varying degrees of current symptom severity. We hypothesized that there would be a negative correlation between symptom severity and valence ratings for memories retrieved to positive cues, such that *higher* depression symptom scores would be related to *less negative* valence ratings for memories retrieved to positive cues.

### Method

#### Participants

We recruited 37 participants with a diagnosis of recurrent MDD. MDD diagnosis and other Axis I comorbidity, according to the *Diagnostic and Statistical Manual for Mental Disorders* (*DSM–IV–TR*; American Psychiatric Association, 2000), were determined using the Structured Clinical Interview for the *DSM–IV* Axis I (SCID-IV; First, Spitzer, Gibbon, & Williams, 2002) by A. J. G. Prior reliability training on the presence of Axis I diagnoses in the SCID-IV, between A. J. G. and T. D., with 28 separate patients resulted in complete agreement. Degree of depression remission was determined using the clinician-rated 21-item Hamilton Depression Rating Scale (HAM-D; Hamilton, 1960), which also served as our continuous measure of depression severity for the correlational analyses. Participants were included in the study if they scored ≥ 7 on the HAM-D—a widely used cutoff (Frank et al., 1991) indicative of clinically significant residual symptoms. Additional exclusion criteria were a current diagnosis of substance dependence and a history of psychosis or organic brain injury. No participants were excluded on these bases.

#### Materials and measures

##### Negative Autobiographical Memory Task (NAMT)

Participants were presented with 16 cue words displayed separately on laminated cards, in a new random order for each participant. Half were positive in valence (*joy, smile, faithful, lively, cheer, lucky, excited, pleasant*) and half were negative (*sad, misery, ashamed, weakness, solemn, tired, upset, bored*). These words were taken from Brittlebank, Scott, Williams, and Ferrier (1993) and are widely used as cues in autobiographical memory studies (Williams et al., 2007). The two valence sets were matched for emotionality (positive: *M* = 4.86, *SD* = 0.86; negative: *M* = 4.85, *SD* = 1.23) and frequency (positive: *M* = 27.75, *SD* = 16.80; negative: *M* = 25.75, *SD* = 16.21; Kucera & Francis, 1967). In response to each word cue, participants were given up to 1 min to recall a memory of a specific, personal event that had happened in their life. Participants were told that the memory they recalled could be something that happened recently or a long time ago. Most important, it was heavily emphasized that the memory they recalled should be *of an unhappy event* (i.e., one that made them feel negative emotions at the time it occurred) even if the cue word itself was positive. Participants were given examples of the ways in which positive cues might link to negative memories; for example, events that had been expected to be positive but turned out to be negative in valence, or events that others experienced positively but where the participants felt otherwise. Only after participants fully understood the nature of the task did the study proceed.

Participants were then given a practice of three neutral words (*wildlife, bread, search*) and feedback was provided. Participants verbalized their responses, which were audio-recorded. If participants’ responses were not personal memories (e.g., they were “categoric” responses; see below), they were prompted until either a personal memory was generated or the 1-min time period expired (when a “no-memory” response was recorded). The experimenter wrote down a brief description of each memory retrieved. It is important that these brief descriptions were only a few words designed to minimally describe the event sufficiently for the participant to know which memory was being referred to (e.g., “Christmas 2014”; “in the kitchen with John last week”; “first day at new job”) and did not include any reference to the emotional valence of the event or the cue that prompted the memory, to circumvent bias. On completion of this part of the NAMT, participants were asked to recall the original instructions they were given—critically, that memories for negative specific events were required to all of the word cues—to ensure that inability to remember task instructions did not influence results. All participants were able to recall the instructions entirely correctly. NAMT performance may have also been influenced by the tendency to recall categoric memories in response to cue words. This tendency is associated with both impaired executive control and depression (Williams et al., 2007). Thus, memories were also transcribed and later coded as specific (discrete events), categoric (summaries of a set of related events), or extended (an event which lasted for longer than one day, e.g., a holiday). Coding was carried out by A. J. G. A random sample of 20% of memories was blind second rated by T. D., with 100% agreement. Finally, experimenters administering the NAMT were kept blind to study hypotheses to protect against unconscious bias.

The recalled memories were subsequently rated by participants in terms of their recollected experienced hedonic valence of the event at the time that it occurred. After the recall task, the aforementioned brief descriptions of the memories (without the accompanying cue words) were presented to participants and they were asked to provide a valence rating of the original experience in terms of how happy or unhappy they felt at the time it occurred on a 7-point scale (method taken from Clark & Teasdale, 1982) from 1 (*extremely unhappy*), through 4 (*neither happy or unhappy*), to 7 (*extremely happy*).

The focus of analyses was on (a) mean valence ratings for memories retrieved to positive cues and (b) the difference between these ratings and the mean valence ratings for memories retrieved to negative cues (mean rating of memories to positive cues minus mean rating of memories to negative cues). These two measures allow us to look at effects of our task manipulation on both absolute and relative valence ratings. We also conducted exploratory analyses on the number of overtly positive memories (i.e., memories rated as 5 or above on our 7-point scale) retrieved to positive cues.

##### Validation of the NAMT

Prior to the clinical study we conducted a preclinical pilot of the NAMT in a small dysphoric sample (*N* = 16; Beck Depression Inventory; BDI; Beck, Rush, Shaw, & Emery, 1979; scores > 10, range 11–40, *M* = 20.25, *SD* = 8.24). Descriptive data are presented in Table S1 (in the Supplemental Material available online). Participants overall rated memories retrieved to positive cues as more positive (less negative) than memories retrieved to negative cues, indicating an effect of context, *t*(15) = 2.99, *p* = .01, *d* = 1.07. More important, participants with higher BDI scores rated memories retrieved to positive cues as relatively more positive (less negative) compared with those with lower BDI scores, *r*(14) = .50, *p* = .04, and higher BDI scores were related to larger discrepancy scores between valence ratings for memories to positive versus negative cues, *r*(14) = .54, *p* = .04. There was no significant association between BDI scores and ratings of memories to negative cues, *r*(14) = –.22, *p* = .40.

The data from this pilot study indicate that increasing levels of depression symptom severity in a subclinical sample are indeed counterintuitively associated with less negatively valenced recollection to positive word cues. This provided a solid justification to take the NAMT to a clinical sample to investigate the hypothesis outlined in the introduction.

#### Procedure

Ethics approval was granted by the NHS East of England National Research Ethics Committee. Following provision of informed consent, participants completed a diagnostic assessment comprising the SCID-IV, HAM-D, and the Spielberger Trait Anxiety Inventory (Spielberger, Gorsuch, Lushene, Vagg, & Jacobs, 1983), which is not reported on here. The NAMT was individually completed one week later.

### Results

#### Description of the sample

According to the SCID-IV, of the 37 volunteers with a diagnosis of recurrent MDD, 21 met criteria for a current major depressive episode (MDE). Of these, 2 were in the “moderately depressed” range (14–18), 6 were in the “severely depressed” range (19–22), and 13 were in the “very severely depressed” range (scores > 22) of the HAM-D (Frank et al., 1991; Hamilton, 1960). The remaining 16 participants did not meet criteria for a current MDE but reported significant residual symptoms according to the HAM-D, 9 with scores in the “mildly depressed” range (8–13), 6 in the “moderately depressed” range, and 1 in the “very severe” range. We also assessed other Axis I diagnoses on the SCID-IV (although data for 4 participants were not available). Of those participants meeting criteria for a current MDE, current comorbid disorders were posttraumatic stress disorder (PTSD; *n* = 6), generalized anxiety disorder (*n* = 5), specific phobia (*n* = 3), social phobia (*n* = 3), obsessive compulsive disorder (*n* = 2), panic disorder (*n* = 1), hypochondriasis (*n* = 1), agoraphobia (*n* = 1), and bulimia nervosa (*n* = 1). Of those participants without a current MDE, only one participant also met the criteria for another current diagnosis—alcohol dependence. There was however significant morbidity in terms of past (i.e., lifetime) diagnoses according to the SCID-IV in this remitted subsample, which were alcohol dependence (*n* = 3), PTSD (*n* = 2), social phobia (*n* = 1), agoraphobia (*n* = 2), substance abuse (*n* = 1), bulimia nervosa (*n* = 2), and anorexia nervosa (*n* = 1).

#### Performance on the NAMT

Demographics and descriptive data for the study measures are presented in Table 1. For the individual differences analyses pertaining to our central hypothesis, zero-order correlations revealed significant associations between valence ratings and both age, *r*(35) = .33, *p* = .04, and educational level, *r*(35) = –.46, *p* = .004. Analyses pertaining to our specific hypotheses therefore used partial correlations with age and educational level as covariates.

**Table 1.** Participant Demographics, Performance on the Negative Autobiographical Memory Task (NAMT), and Measures of Depressive Symptoms for Study 1 (*N* = 37)

Variable	*M* (*SD*)
Hamilton Depression Rating Scale Score	20.73 (8.24)
Gender (female), *n* [%]	21 [57]
Age (years)	49.62 (12.22)
Education (*n*)	8, 8, 11, 6, 4^a^
Valence rating to positive cues	2.87 (1.49)
Valence rating to negative cues	1.72 (0.43)
No. of positive memories to positive cues [%]	1.38 (2.02) [17]
No. of positive memories to negative cues [%]	0.08 (0.36) [1]
No. of “no memories” to positive cues [%]	1.46 (1.64) [18]
No. of “no memories” to negative cues [%]	0.62 (0.95) [8]
No. of “categoric” memories to positive cues [%]	1.03 (1.50) [13]
No. of “categoric” memories to negative cues [%]	1.22 (1.44) [15]
No. of “extended” memories to positive cues [%]	0.59 (0.76) [7]
No. of “extended” memories to negative cues [%]	0.59 (0.93) [7]

a Education (U.K. system) = numbers of General Certificate of Secondary Education, advanced levels, further education diploma, higher education bachelor’s degree, postgraduate qualification (quantified as a linear continuous variable for the purpose of analyses), respectively.

Overall, participants rated memories retrieved to positive cues as less negative than memories retrieved to negative cues, *t*(36) = 5.03, *p* < .01, *d* = 1.05. Examination of numbers of overtly positive memories recalled (those rated 5 or above on our 7-point valence scale) revealed that participants retrieved more such memories in response to positive cues (*n =* 51, 17.2%), compared with negative cues (*n =* 3, 1%), Wilcoxon’s *t* = 3.38, *p* < .01. HAM-D scores were not significantly associated with the number of overtly positive memories retrieved to positive cues, *r*_s_(35) = .20, *p* = .24.^1^

As hypothesized, higher scores on the HAM-D were significantly associated with the degree to which memories retrieved to positive cues received more positive/less negative valence ratings, *pr*(33) = .36, *p* = .03, and with a greater difference between valence ratings for memories to positive versus negative cues, *pr*(33) = .37, *p* = .03. These results remained the same when partialling out the number of overtly positive memories recalled to positive cues, positive cues; *pr*(32) = .46, *p* = .007, difference; *pr*(32) = .46, *p* = .007. There was no such significant association between HAM-D scores and valence ratings of memories retrieved to negative cues, *pr*(33) = –.03, *p* = .86. Again, these results remained the same when partialling out the number of overtly positive memories recalled to negative cues, *pr*(32) = –.01, *p* = .94.

Few of the memories retrieved on the NAMT were coded as “categoric” or “extended” (Table 1) and there was no difference as a function of cue word valence, *t* < 1. This was likely due to the efforts in prompting participants to recall specific memories during the task (and was mirrored in the pilot study; see Table S1). There were, however, more “no memories” recorded to positive cues relative to negative cues, *t*(36) = 3.28, *p* = .002, although numbers overall were small (Table 1). There was a weak trend for a correlation between the number of no memories generated and depression severity, *r*(35) = 0.29, *p* = .08. However, our profile of results remained the same and significant when partialling out numbers of “no memories” and the “no memories difference score,” respectively: HAM-D with valence to positive cues, *pr*(32) = .34, *p* = .05; HAM-D with differential valence ratings, *pr*(32) = .37, *p* = .03.

### Discussion

These findings support our hypothesis that affective context plays an important role in overriding retrieval goals in depression. The data showed that increasing levels of depression symptom severity were counterintuitively associated with less negatively valenced recollection to positive word cues during the NAMT. These results suggest that the more depressed participants were less able to override the biasing influence of the positive cue words (presumably because of relatively impaired executive control), even though they had both putative mood-congruent memory biases and the explicit task instructions working in favor of retrieving negative memories.

## Study 2: Performance on NAMT as a Function of Individual Differences in Working Memory Capacity

Our thesis is that executive control deficits associated with increasing severity of depression are a strong candidate for what impairs the ability to override the priming effects of context when retrieving autobiographical memories. The results from Study 1 are consistent with this view, and consequently raise the question of whether individual differences in executive control would modulate this effect in the general population. We therefore administered the NAMT in an unselected community sample, and examined whether performance on the NAMT varied as a function of individual differences in working memory capacity (WMC)—a widely employed index of executive control in the domain of memory (Unsworth & Engle, 2007). We hypothesized that, as in Study 1, across all participants valence ratings for memories recalled to positive cues would be less negative than memories recalled to negative cues. We also predicted that the ability to override contextual cues would be influenced by individual differences in WMC, such that *poorer WMC* would be associated with *less negative* valence ratings for memories retrieved to positive cue words. As depressive symptoms were not related to the number of overtly positive memories recalled in our exploratory analyses in Study 1, we did not have a clear a priori prediction regarding the relationship between WMC and the number of overtly positive memories recalled.

### Method

#### Participants

A total of 63 participants was recruited from the MRC Cognition and Brain Sciences Unit Volunteer Panel—a database of approximately 2,000 community volunteers who have agreed to help with psychological research, recruited to the panel via advertisements in local newspapers and online. To be eligible for the study participants had to be fluent in English and over 18 years of age.

#### Materials and measures

##### Negative Autobiographical Memory Task (NAMT)

The NAMT was administered exactly as described in Study 1.

##### The Operation Span Test (OSPAN; Unsworth, Heitz, Schrock, & Engle, 2005)

WMC was indexed using the OSPAN task. The core element of the OSPAN task involves presenting participants with a neutral to-be-remembered word on a card, for 2 s, followed by a simple numeric equation (e.g., (9 ÷ 3) – 2 = 1) for 4 s. Participants are required to verify whether the answer to the equation is correct or incorrect. After a series of such word-verify pair elements, participants are asked to recall all of the words they have seen in the order in which they were presented. The OSPAN consists of 12 trials, 3 comprising two word-verify pairs, 3 comprising three pairs, 3 comprising four pairs, and 3 comprising five pairs. Participants who verified fewer than 85% of the mathematical equations successfully have their data set aside but this did not apply to any participant in the present study. A trial is scored as correct if a participant recalls all of the to-be-remembered words (ranging from 3 to 5 words) in the presented order. For these correct trials only, the numbers of to-be-remembered words correctly recalled are summed across the whole task and the sum divided by the total number of words presented, giving a score between 0 and 1. This is known as all-or-nothing load scoring (A. R. A. Conway et al., 2005) and has been shown to be sensitive to individual differences in cognitive-affective processing (e.g., Schweizer & Dalgleish, 2011).

##### Beck Depression Inventory (Beck et al.,1979)

The BDI was also administered to index any depressive symptoms in this unselected sample (cf. the NAMT pilot study).

#### Procedure

Following provision of informed consent participants completed the tasks and measures individually and face-to-face with the experimenter, in a quiet testing room. Participants first completed the OSPAN followed by the NAMT and BDI.

### Results and discussion

Descriptive statistics for sample characteristics and performance on the NAMT are presented in Table 2. In line with our first hypothesis, participants overall rated memories retrieved to positive cues as less negative than memories retrieved to negative cues, *t*(62) = 4.92, *p* < .001, *d* = 0.79. In terms of the number of overtly positive memories retrieved, participants also retrieved more overtly positive memories (> 4 on the 7-point scale) in response to positive cues (total of *n* = 37, 7.3% across all subjects), compared with negative cues (total of *n* = 3, 0.6% across all subjects), Wilcoxon’s *t* = 2.69, *p* = .01. The difference in ratings across all participants for memories to positive versus negative cues remained significant when covarying for the number of overtly positive memories retrieved in the two conditions, *F*(1, 60) = 20.70, *p* < .001, *d* = 0.82.

**Table 2.** Participant Demographics, Performance on the Negative Autobiographical Memory Task (NAMT), and Measures of Depressive Symptoms and Working Memory Capacity for Study 2 (*N* = 63)

Variable	*M* (*SD*)
Gender (female), *n* [%]	46 females [73]
Age (years)	42.49 (13.71)
Education (*n*)	8, 20, 8, 19, 8^a^
OSPAN score	0.58 (0.19)
BDI score	6.69 (6.24)
Valence rating to positive cues	2.48 (1.04)
Valence rating to negative cues	1.84 (0.43)
No. of positive memories to positive cues [%]	0.59 (1.64) [7]
No. of positive memories to negative cues [%]	0.05 (0.21) [0.6]
No. of “no memories” to positive cues [%]	1.81 (1.87) [22]
No. of “no memories” to negative cues [%]	0.63 (0.99) [7]
No. of “categoric” memories to positive cues [%]	0.37 (0.68) [4]
No. of “categoric” memories to negative cues [%]	0.33 (0.65) [4]
No. of “extended” memories to positive cues [%]	0.37 (0.73) [4]
No. of “extended” memories to negative cues [%]	0.48 (0.80) [6]

Note: BDI = Beck Depression Inventory; OSPAN = Operation Span.

a Education (U.K. system) = numbers of General Certificate of Secondary Education, advanced levels, further education diploma, higher education bachelor’s degree, postgraduate qualification, respectively.

In line with our second hypothesis, scores on the OSPAN were significantly associated with the degree to which memories retrieved to positive cues received less negative valence ratings, *r*(61) = –.25, *p* = .05, with participants with lower WMC scores rating memories as relatively less negative. This effect was no longer significant once the number of overtly positive memories was partialled out, *r*(60) = –.11, *p* = .41. There was also a trend for a significant relationship between OSPAN scores and the difference between ratings to memories for positive versus negative cues, *r*(61) = –.23, *p* = .07, with lower OSPAN scores associated with differentially less negative ratings to positive cues, but again this effect disappeared when controlling for the number of overtly positive memories, *r*(58) = –.06, *p* = .66. There was no significant association between OSPAN scores and valence ratings of memories retrieved to negative cues, *r*(61) = –.06, *p* = .63.

Interestingly, OSPAN scores were also significantly associated in nonparametric analyses with the number of overtly positive memories retrieved to positive cues, *r*_s_(61) = –.26, *p* = .04, with those scoring lower on the OSPAN retrieving more positive memories. There were insufficient positive memories retrieved to negative cues to conduct comparable analyses.

Very few of the memories retrieved were coded as “categoric” or “extended” (Table 2), and this did not differ as a function of cue valence, *t* < 1. Numbers of “no memories” were similarly small but did differ as a function of cue valence, *t*(62) = 6.05, *p* < .001, *d* = 1.08. Repeating the above analyses with numbers of “no memories” in the relevant valence conditions covaried made no difference to the pattern of findings.

In this unselected sample, BDI scores were not significantly associated with scores on the OSPAN, *r*(61) = .02, *p* = .88, or with valence scores for memories retrieved to positive, *r*(61) = .20, *p* = .11, or negative cues, *r*(61) = .21, *p* = .10. This is unsurprising given that the majority of participants (89%) were in the normal range on the BDI and the modal score was zero.

Finally, we completed a post hoc comparison of NAMT performance between the 21 individuals from our clinical sample with MDD who were *currently experiencing* an acute episode of depression (the depressed group) and 22 individuals from the Study 2 community sample with the lowest self-reported depression scores (BDI scores < 4; the control group). These analyses indicated that the depressed group (*M* = 1.52, *SD* = 1.97) reported a significantly greater number of overtly positive memories, *t*(30.71) = –2.57, *p* = .02, *d* = 0.75, compared with the control group (*M* = 0.27, *SD* = 1.08). The depressed group also tended to rate memories as more positive following positive cues (*M* = 3.09, *SD* = 1.62) than controls (*M* = 2.31, *SD* = 0.74), *t*(27.73) = 2.03, *p* = .05, *d* = 0.62, but also tended to rate memories retrieved to negative cues as more negative (*M* = 1.66, *SD* = 0.38) than controls (*M* = 1.93, *SD* = 0.48), *t*(41) = 2.00, *p* = .05, *d* = 0.61. This reinforces the conclusion that depression is associated with a tendency to be differentially influenced by the presence of positive cues, in line with Study 1.

In sum, Study 2 showed that in a healthy community sample, as in our clinical sample in Study 1, valence ratings of memories retrieved to positive cues were less negative than those accorded to memories retrieved to negative cues. There were also more overtly positive memories retrieved to positive cue words relative to negative cues. It is important that, as anticipated, WMC scores moderated these effects. Lower WMC was significantly associated with less negative valence ratings of personal memories retrieved to positive cue words, supporting our hypothesis, and with the retrieval of more overtly positive memories to those cues. This effect was not significant once the number of overtly positive memories were accounted for in post hoc analyses, suggesting that at least for a community sample, executive control may be more important in preventing explicit errors during the NAMT, rather than impacting valence ratings per se. However, care is needed in interpreting this null result as it may also be a function of insufficient range within the ratings scores once the overtly positive scores were removed and a consequent lack of power in these post hoc analyses.

## General Discussion

We developed a laboratory task—the Negative Autobiographical Memory Task (NAMT)—with the principal aim of highlighting how depression severity influences susceptibility to contextual priming effects (Study 1). In line with our prediction, we found that, when instructed to retrieve negative personal memories in response to positive cue words, clinically depressed individuals with *more* severe depressive symptoms actually retrieved memories that were rated as *less* hedonically negative than those retrieved by less severely depressed peers. This finding of a mood-incongruent effect in autobiographical recollection, alongside similar results in our pilot study with a dysphoric sample, provide important support for the significant influence of contextual priming effects in dysphoria and depression.

A strong candidate account of these results is that the more impoverished executive control associated with higher levels of acute depressive symptoms (McDermott & Ebmeier, 2009) means that fewer executive resources are available to override the priming effect generated by the positive word cues. As noted in the introduction, such priming would bias the autobiographical search process toward more positive responses that therefore contravene the task instructions to generate negative memories. Poorer executive control would compromise the ability to override the effects of this primed bias to generate task-appropriate memories. This explanation is more compelling than any account involving underlying (context-free) memory biases in depression. Evidence suggests that such fundamental biases are generally in favor of negative material (Blaney, 1986), rather than positive, and such effects would also be expected to reveal themselves independent of context, that is, in both the positive and negative cue conditions, which was not the case here.

This candidate executive control explanation led to a clear prediction which we tested in Study 2—that in a nondepressed community sample, individuals with lower levels of executive control would perform in an analogous way to those with high levels of depressive symptoms in Study 1. Results from Study 2 supported this prediction. Poorer performance on a WMC paradigm—an index of executive control ability—was associated with less negative valence ratings, and with the generation of a greater number of overtly positive memories, to positive cues. Together these findings suggest that the core aspect of elevated depressive symptomatology that drives this context sensitivity effect is relatively impoverished executive control.

The current experimental set-ups were contrived to highlight this phenomenon and clearly day-to-day cognition would rarely, if ever, involve actively seeking to retrieve negative memories in response to positive contextual cues. However, the same susceptibility to contextual priming demonstrated here is likely to have a daily impact in the obverse condition when those with depression, for instance, seek to recollect neutral or positive information in the context of negatively valenced intrinsic (e.g., thoughts and ruminations) or extrinsic (e.g., environmental) contexts. We did not design the current experiment around this obverse scenario as it would have been difficult to exert experimental control over the effects of intrinsic negative context (e.g., depressive mood, depressogenic thoughts, rumination) in those with depression. The current data therefore have potential clinical implications, in terms of novel intervention targets for depression. One possibility is that augmenting the ability to ignore maladaptive emotional contexts through some form of executive control training could be advantageous to sufferers of depression, as it would permit more effective regulation of any detrimental effects of intrinsic and extrinsic emotional contextual primes (e.g., Schweizer, Hampshire, & Dalgleish, 2011).

As valence ratings were not as negative for memories that were retrieved to a positive cue, our data also imply that those with depression should be able to benefit from the priming effects of positively valenced contexts. This suggests that scaffolding autobiographical recollection with relevant positive mnemonic cues may have some benefit (Dalgleish & Werner-Seidler, 2014). Current autobiographical memory training programs for those with depression make use of emotionally positive or benign cues to guide autobiographical retrieval (e.g., Hitchcock et al., 2016), and our results support the rationale that improving the ability to retrieve positive memories in response to positive cues may have beneficial effects for overwriting the automaticity toward negative autobiographical information (see Hitchcock, Werner-Seidler, Blackwell, & Dalgleish, 2017, for a review).

An issue across both Study 1 and Study 2 that merits consideration is whether the findings simply result from demand effects influencing the ratings of the remembered experiences in a less negative direction when the cue words are positive, rather than the retrieval process itself generating memories of genuinely less negative experiences. The former possibility seems unlikely because in the vast majority of cases (over 80%) the ratings were made with respect to overtly negative memories (as highlighted in the results section); the memory ratings were solicited after the retrieval component of the task and were made in response to a brief descriptor of the memory that did not include any detail of the emotional valence of the event; and the ratings were made in the absence of the original cue words. Furthermore, with respect to Study 1, there is considerable evidence that response bias effects when making valence ratings tend in the direction of more negative ratings as depression increases (Zuroff, Colussy, & Wielgus, 1983) and would thus work against the grain of our key Study 1 findings.

A related question concerns the overtly positive memories that are recollected on the task. Is it the case that participants temporarily “forgot” the task instructions in these instances? We know that all participants were able to correctly recall the instructions once the task was completed but this does not preclude some form of acute “goal neglect.” It is worth noting that the task instructions never varied—they were always to recall negative memories—so it seems unlikely that participants were not clear, even temporarily during the task, about what they should be doing. Given this, allied to the fact that participants unanimously reported the instructions back correctly after the task, we are confident that, had we probed participants at any time during the study, they would have been able to tell us what they should have been doing. What seems more likely is that the context (positive cues) is biasing the search of the autobiographical memory database toward positivity as we discuss in the introduction. In some cases this search will generate overtly positive memories as candidate responses. The participant’s job in such instances is to check those memories against the explicit search requirements and reject them and continue the search for a bona fide negative memory. In a small number of instances, memories that we as experimenters later label as overtly positive on the basis of the participant’s ratings are not rejected as inappropriate (because of either higher symptoms of depression and/or poor executive control) and are given as responses on the task.

It is important to note that the sample size for Study 1 was modest for a correlational design. However, we were able to demonstrate consistent effects across a dysphoric and a clinically depressed sample, and the effects observed in the nonclinical Study 2 with a larger sample support the reliability of the findings.

In summary, the current findings highlight the marked susceptibility to contextual cues in depression (mirrored by those with low levels of executive control) and suggest that systematic cognitive training programs targeted at enhancing the ability to ignore disadvantageous affective contexts (e.g., Schweizer et al., 2011), or using autobiographical memory training techniques to scaffold recollection using positive cues (Hitchcock et al., 2017) may reap clinical benefits.

## Supplementary Material

Supplementary material

## Footnotes

1. Note that age and educational level were not covaried for this analysis because of the need for a nonparametric approach necessitated by the small numbers of positive memories retrieved.
