# Supplementary material for: The Impact of Affective Context on Autobiographical Recollection in Depression
Source: Clin Psychol Sci. 2017 Nov 16;6(3):315–24. doi: 10.1177/2167702617740672 (PMC5952300; doi:10.1177/2167702617740672)
Supplement: Supplementary material [file DalgleishSupplementary_Materials.pdf]

Supplementary Table 1

Participant characteristics and task performance during pilot of the Negative Autobiographical Memory Task (N = 16)

	Mean (SD)
Gender	12 Females
Age	30.38 (10.88) years
Beck Depression Inventory	20.25 (8.24)
Valence rating to positive cues	2.57 (1.08)
Valence rating to negative cues	1.68 (0.44)
No. of positive memories to positive cues	0.63 (1.36)
No. of positive memories to negative cues	0
No. of ‘no memories’ to positive cues	0
No. of ‘no memories’ to negative cues	0
No. of ‘categoric’ memories to positive cues	0
No. of ‘categoric’ memories to negative cues	0
